# Integrated Omics Analysis Revealed the Differential Metabolism of Pigments in Three Varieties of *Gastrodia elata* Bl

**DOI:** 10.3390/ijms262411839

**Published:** 2025-12-09

**Authors:** Xiaohua Li, Huaijing Zhu, Bingbing Zhang, Dahui Liu

**Affiliations:** 1 School of Pharmacy, Hubei University of Traditional Chinese Medicine, Wuhan 430065, China; lixiaohua2007@hotmail.com (X.L.);; 2 Hubei Shizhen Laboratory, Wuhan 430065, China

**Keywords:** *Gastrodia elata*, metabolomics, transcriptomics, carotenoids, regulation mechanism

## Abstract

*Gastrodia elata* Blume is a well-known traditional Chinese medicine. The color of flower and flower stalk are important characteristics in the classification of *G. elata*. However, the mechanisms of pigment formation in different types of *G. elata* are not yet elucidated. To understand this, targeted metabolomics as well as transcriptomics analyses were carried out in this study. The differential accumulation and the typical components of pigments in different types of *G. elata* were elucidated. According to our research, the accumulation of carotenoids rather than anthocyanins likely contributes to the pigment content in *G. elata*. The different accumulations of carotenoids including violaxanthin, lycopene, α-carotene, and α-cryptoxanthin are the main reasons that contribute to the color differences in the flowers and flower stalks of these three *G. elata* varieties. Integrated multi-omics analysis enriched 50 and 17 differential genes in the flavonoid–anthocyanin and carotenoid biosynthesis pathways, respectively. Among these, *PSY*, *PDS*, *CCD*, *UGT*, and *ANR* were identified as critical genes responsible for the differential pigment accumulation in *G. elata* varieties, while the MYB TFs were tightly associated with main genes expression and content of carotenoids. Overall, this study enhances the current understanding of pigments’ metabolites profiles and contributes valuable insights into the molecular mechanisms underlying *G. elata* carotenoid biosynthesis; these findings also provide valuable guidance for future carotenoid biofortification and molecular breeding in *G. elata*.

## 1. Introduction

The pigment compounds are widely distributed in the plant kingdom, and primarily consist of porphyrins, carotenoids, flavonoids, and betalains. These phytochemicals play vital roles in plant growth and development; notably, carotenoids and anthocyanins are integral to processes including photoprotection and stress mitigation, and are equally pivotal in generating the visual and olfactory cues that attract animal pollinators and seed dispersers [1,2,3]. Serving as natural antioxidants, plant pigments possess diverse biological functions and pharmacological activities, including antioxidative and anti-inflammatory effects, as well as risk reduction for cardiovascular diseases, cancer, hypertension, and hyperlipidemia; these compounds demonstrate significant therapeutic and preventive potential in disease management [2,4,5]. In recent years, the study of pigment substances has become a hot topic in the research area. Extensive research has been conducted on pigment constituents and their biosynthetic pathways in crops as well as horticultural plants, with anthocyanins and carotenoids emerging as the predominant phytopigments that significantly influence nutritional composition and product quality [1].

Elucidating the genetic basis of pigments and breeding novel varieties enriched with these phytochemicals has become a principal objective in modern breeding programs. This approach is also conducive to enhancing nutritional quality and improving ornamental traits in crops or horticultural species. The biosynthetic pathways and regulatory mechanisms governing these pigments have been extensively elucidated through integrated multi-omics approaches across diverse plant systems; multiple genes and transcription factors regulating the biosynthesis of pigment constituents have been identified [2]. Using integrated multi-omics approaches including genomics, transcriptomics, metabolomics, pangenomics, and genome-wide association studies (GWAS), researchers can establish a comprehensive understanding of the regulatory genomic machinery underlying key quality traits in tea, particularly, that the comprehensive understanding of anthocyanin biosynthesis facilitates the breeding of anthocyanin-rich tea cultivars [3]. Zeng et al. successfully reconstructed the anthocyanin biosynthesis pathway in rice endosperm by expressing four rice endogenous genes, yielding rice grains enriched with anthocyanins [4]. Zhu et al. generated a novel biofortified germplasm “Purple Endosperm Rice” by engineering anthocyanin biosynthesis in the endosperm with a high-efficiency transgene stacking system [5]. Sun et al. efficiently engineered high-level biosynthesis of anthocyanins in the peel and flesh of tomato by expression of a functional *SlAN2-like* gene driven by the fruit-specific promoter in a tomato cultivar [6]. CRISPR technology was successfully applied to enhance the biosynthesis and accumulation of pigmentation in crops, fruits, vegetables, and ornamental plants [7].

*Gastrodia elata* Bl., also well known as “Tian ma”, belongs to the *Orchidaceae* family, and the dried tubers of *G. elata* have been traditionally used as medicine and food for more than a thousand years in Asian countries. *G. elata* exhibits remarkable therapeutic efficacy, incerebrovascular protection, neuroprotection, antidepressant activity, anticonvulsant applications, and so on [8,9]. Based on the pigment of floral and stalks, tuber morphology, and water content characteristics, *G. elata* is classified into several varieties in China, while the phenotype of red-stalk original varieties (*G. elata* Bl. f. elata, GR), along with brown-stalks (*G. elata* Bl. f. g1auca, GB) and green-stalk (*G. elata* Bl. f. viridis, GG) varieties are commonly artificial cultivated in China due to the predominant production or environmental resistance [8]. Current research on *G. elata* primarily focuses on phenolics components and pharmacological activities [9]. Although coloration serves as a crucial characteristic for *G. elata* classification, the composition of color substances and the regulatory mechanisms of color-related metabolites in different *G. elata* varieties remain unclear.

Recent advancements in analytical technologies, particularly chromosome-level genome data and integrated multi-omics approaches, have enabled comprehensive investigations into metabolites regulation mechanisms in *G. elata* [8,9]. However, the complete understanding of the composition and the molecular mechanism of pigments at genetic-biological and molecular levels remains unclear in *G. elata*. In our study, we integrated multi-omics analysis to conduct the pigments’ metabolic profiling of *G. elata* three varieties, along with integrated analysis of the metabolome and transcriptome data. In all, our study elucidates the material basis underlying color variations among different varieties of *G. elata*, we reveal valuable insights into the molecular mechanisms underlying *G. elata* carotenoid biosynthesis and provide valuable guidance for future carotenoid biofortification and breeding in *G. elata*.

## 2. Results

### 2.1. Transcriptome Sequencing and Differential Gene Expression Analysis in Different Varieties of G. elata

**Figure 1:**
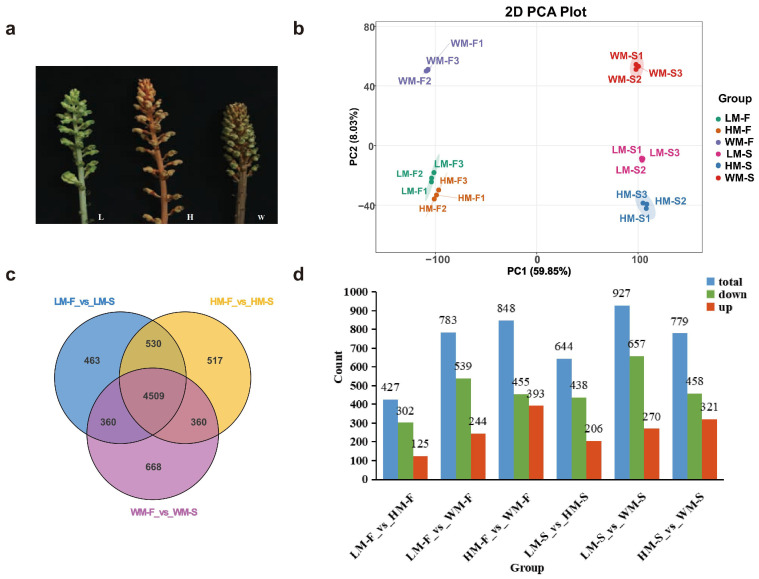
Phenotypes and transcriptome profiles of three different varieties of *G. elata*. (**a**) The phenotypic images of three varieties of *G. elata*. LM (L), HM (H), WM (W); (**b**) The Score plot of principal component analysis (PCA) of genes; (**c**) Venn diagram of transcriptome data between different comparison groups; (**d**) bar chart of differential expression genes, with red indicating up-regulated genes and green indicating down-regulated genes in *G. elata* varieties.

**Figure 2:**
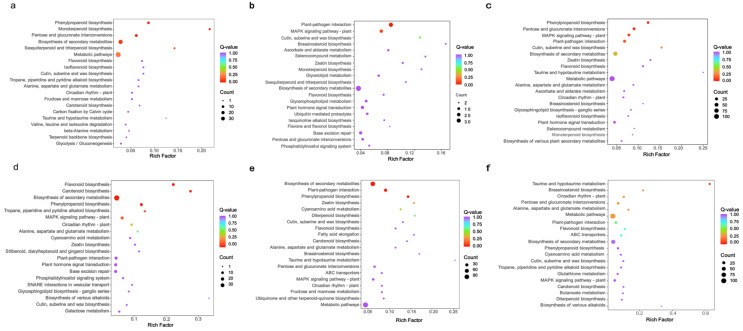
KEGG enrichment analysis of differential expression genes in *G. elata* varieties. KEGG enrichment in HM-F vs. LM-F (**a**), WM-F vs. LM (**b**), WM-F vs. HM-F (**c**), HM-S vs. LM-S (**d**), WM-S vs. LM-S (**e**), WM-S vs. HM-S (**f**) comparisons. The *x*-axis represents the Rich Factor, the *y*-axis shows pathway names, and the color intensity of the bubbles corresponds to represent Q-value of enrichment, with deeper red indicating higher significance. Bubble size reflects the number of DEGs enriched in each pathway.

Advances in analytical technologies have made multi-omics approaches instrumental in deciphering metabolic pathways in plants. These integrated strategies now provide key methodologies and foundational insights for investigating coloration-related substances and unraveling their biosynthetic mechanisms in medicinal plants [10]. To explore the internal mechanism of differential metabolites accumulation, we conducted RNA-seq for three colors of *G. elata* flowers (F) and flower stalks (S), as shown in Figure 1a. In total, 973.81 million clean reads with an average of 54.10 million per sample were generated. The Q30 ratio of each sample was 93.50~94.08%, and the mapping ratio of reads to reference genome was 98.34~98.70%. Principal component analysis (PCA) was performed, and results of the samples revealed a clear separation among groups (Figure 1b). The flowers and flower stalks of different varieties obtained good results in the two components. Through transcriptome data comparison, a total of 6991 differentially expressed genes (DEGs) were identified. Among these, 4509 genes were commonly detected across the three comparisons, namely, LM-F vs. LM-S, HM-F vs. HM-S, and WM-F vs. WM-S. The number of DEGs in WM-F vs. WM-S was significantly higher than that in the other two groups (Figure 1c). Specifically, a total of 427 DEGs were identified between LM-F and HM-F, comprising 125 up-regulated and 302 down-regulated genes. The comparison of LM-F to WM-F yielded 783 DEGs, comprising 244 up-regulated and 539 down-regulated, while that of HM-F to WM-F yielded 848 DEGs, comprising 455 up-regulated and 393 down-regulated. Among the S groups, LM-S versus HM-S showed 644 DEGs, of which 206 were up-regulated and 438 down-regulated. LM-S versus WM-S showed 927 DEGs, of which 270 were up-regulated and 657 down-regulated. Lastly, HM-S versus WM-S showed 779 DEGs, of which 321 were up-regulated and 458 down-regulated, respectively (Figure 1d, Supplementary Table S1). The KEGG enrichment analysis (Figure 2) revealed that DEGs in HM-F vs. LM-F were mainly enriched in metabolic pathways, biosynthesis of secondary metabolites, pentose and glucuronate interconversions, phenylpropanoid biosynthesis (Figure 2a). In WM-F vs. LM-F, DEGs were primarily involved in biosynthesis of secondary metabolites, plant–pathogen interaction, plant hormone signal transduction (Figure 2b). Additionally, metabolic pathways, biosynthesis of secondary metabolites, phenylpropanoid biosynthesis, plant–pathogen interaction, plant hormone signal transduction were also enriched in WM-F vs. HM-F (Figure 2c). DEGs were also similarly enriched in biosynthesis of secondary metabolites and metabolic pathways during flower stalk comparisons (Figure 2d–f).

### 2.2. The Anthocyanin Biosynthesis Pathway in G. elata Varieties

**Figure 3:**
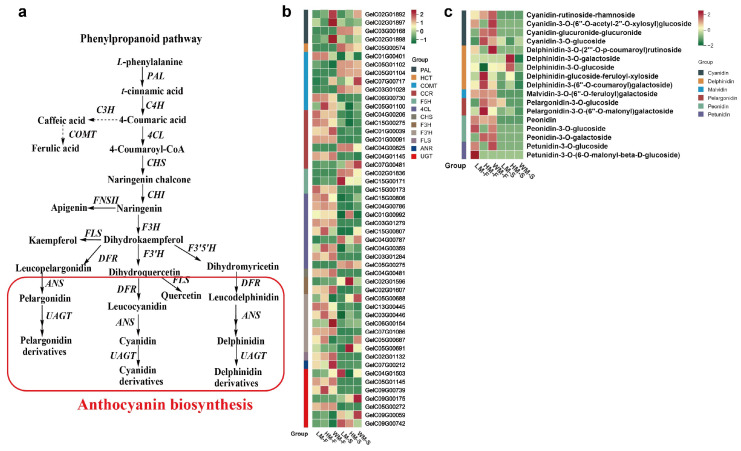
Simplified scheme and heat map of anthocyanin biosynthesis genes in *G. elata* varieties. (**a**), Anthocyanin biosynthesis pathway. PAL, phenylalanine ammonia-lyase; C4H, cinnamate 4-hydroxylase; 4CL,4-coumarate-CoAligase; CHS, chalcone synthase; CHI, chalcone isomerase; F3H flavonoid 3-hydroxylase; F3′H, flavonoid 3′-hydroxylase; F3′5′H, flavonoid 3′5′-hydroxylase; DFR, dihydroflavonol 4-reductase; ANS, anthocyanidin synthase; FLS, flavonol synthase; UGT, UDP-glucose flavonoid 3-O-glucosyltransferase; ANR, anthocyanidin reductase. (**b**), Heat map clustering showing the expression of genes involved in flavonoid and anthocyanin biosynthesis. The color scale represents the log-transformed FPKM value. Darker red indicates high expression, and green indicates low expression. (**c**), Heat map of differential anthocyanin metabolites between *G. elata* varieties, with darker red indicating increased metabolites and green indicating reduced metabolites.

According to DEGs analysis, a total of 1089 differential genes were annotated in metabolic pathways. Through gene annotation analysis, there were fifty DEGs encoding enzymes related to phenylpropanoid–anthocyanin biosynthesis metabolism that were screened, including four *PAL*, nine *4CL*, one *CHS*, and *F3′H*, two *F3H*, there *F5H*, one *FLS*, one *DFR*, one *ANR*, seven *UGT* genes, and seven *CMOT* (Figure 3a,b, Supplementary Table S2). Among them, the expression patterns of *COMT* (*GelC05G01104*), *PAL* (*GelC03G00168*), *F5H* (*GelC02G01836*), *4CL* (*GelC05G00275*), and *HCT* (*GelC05G00574*) were significantly up-regulated in flower stalks than in that of flower, whereas *CHS* (*GelC04G00481*), *4CL* (*GelC03G01284* and *GelC03G01279*), *F5H* (*GelC15G00173*), *UGT* (*GelC05G01145*), *ANR* (*GelC07G00212*), and *FLS* (*GelC02G01132*) were significantly up-regulated in flower than in that of flower stalks; especially, the gene expression levels of ANR were 14.65-, 14.55-, and 8.12-fold higher in flowers of WM, HM, and LM when compared to flower stalks (Figure 3b, Supplementary Table S2).

### 2.3. Metabolomic Analysis of Anthocyanins in G. elata Varieties

The anthocyanin profiling in the different *G. elata* varieties was determined using a UPLC-MS platform with triplicate biological sampling. A total of 18 anthocyanin compounds were identified, including 7 petunidins, 5 delphinidins, 2 malvidin, 5 peonidins, 4 cyanidins, and 2 pelargonidins (Supplementary Table S3). There were significant differences between the flowers and stalks. Flowers contain 5 delphinidins, 4 cyanidins, 3peonidins, showing more kinds of anthocyanins than stalks, and 13 (11 up- and 2 down-regulated), 16 (16 up-regulated), and 13 (12 up- and 1 down-regulated) differentially accumulated anthocyanin metabolites were identified in LM-F vs. LM-S, WM-F vs. WM-S, HM-F vs. HM-S, respectively (Figure 3c, Supplementary Table S3). Totally, WM-F exhibited significantly elevated anthocyanin production versus other varieties. Delphinidin-3-*O*-(2‴-*O*-p-coumaroyl) rutinoside, Cyanidin-rutinoside-rhamnoside, the top two highest anthocyanin metabolites detected from flowers, accumulated with the amount of 0.84 µg/g and 0.45 µg/g in WM-F, 0.39 µg/g and 0.24 µg/g and LM-F, respectively; there was no delphinidin-3-*O*-(2‴-*O*-p-coumaroyl) rutinoside obtained in HM-F along with 0.34 µg/g cyanidin-rutinoside-rhamnoside. Cyanidin-3-*O*-glucoside was only obtained in HM-F and WM-F with the amount of 0.15 µg/g and 0.11 µg/g, while absent in LM-F. A trace amount of 4 delphinidins and 2 pelargonidins were detected in flower stalks with 0.01–0.02 µg/g (Figure 3c, Supplementary Table S3).

### 2.4. The Carotenoid Biosynthesis Pathway and Accumulation in G. elata Varieties

**Figure 4:**
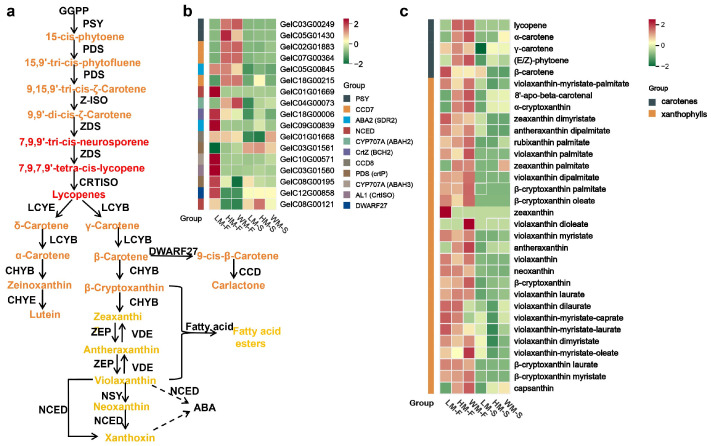
Transcriptional and metabolic profiling of carotenoid accumulation in *G. elata*. (**a**), Schematic of the carotenoid biosynthesis pathway in plants. GGPP, geranylgeranyl diphosphate; PSY, phytoene synthase; PDS, phytoene desaturase; Z-ISO, *ζ*-carotene isomerase; ZDS, *ζ*-carotene desaturase; CRTISO, carotene isomerase; LCYB, lycopene *β*-cyclase; LCYE, lycopene *ε*-cyclase; CHYB, *β*-ring hydroxylase; DWARF27, beta-carotene isomerase; CCD, carotenoid cleavage dioxygenase; ZEP, zeaxanthin epoxidase; NCED, 9-*cis*-epoxycarotenoid dioxygenase; NSY, neoxanthin synthase; ABA, abscisic acid; GA, gibberellic acid. Different font colors indicate variations in the synthesis colors of carotenoids. (**b**), the heat map of genes’ expression levels in the carotenoid biosynthesis pathway of *G. elata*. (**c**), the carotenoid accumulation in *G. elata*.

Carotenoid metabolic pathways have been extensively researched in plants. The central biosynthetic route involves a series of key reactions, including condensation, desaturation, isomerization, hydroxylation, oxidation, and epoxidation, that lead to the production of diverse carotenes and xanthophylls. In plants, these compounds are biosynthesized primarily through the MEP (2-C-methyl-D-erythritol 4-phosphate) pathway, involving sequential enzymatic catalysis by genes such as *DXR* (1-deoxy-D-xylulose 5-phosphate reductoisomerase), *FPPS* (farnesyl pyrophosphate synthase), *PSY* (phytoene synthase), *PDS* (phytoene desaturase), *ZDS* (ζ-carotene desaturase), and *CCD* (carotenoid cleavage dioxygenase) [1] (Figure 4a). To further explore the mechanism of carotenoids biosynthesis in *G. elata*, the expression patterns of enzyme encoding genes in the carotenoid pathways were analyzed. A total of 17 of these genes were differentially expressed in carotenoid biosynthesis between these varieties (Figure 4b, Supplementary Table S4), including two transcripts of *PSY* (*GelC03G00249*, *GelC18G00006*), *PDS* (*GelC01G01668*, *GelC01G01669*), *CYP707A* (GelC09G00839, *GelC04G00073*), *AL1* (*CrtISO*, *GelC08G00195*) and *ABA2* (*GelC05G01430*, *GelC07G00364*), three *CCD7* (*GelC02G01883*, *GelC03G01560*, *GelC03G01561*), and one for *CCD8* (*GelC10G00571*), *DWARF27* (*GelC05G00845*), *NCED* (*GelC08G00121*, *GelC18G00215*), *CrtZ* (*GelC12G00858*). *PSY* (*GelC03G00249*, *GelC18G00006*), *CCD7* (*GelC02G01883*, *GelC03G01560*, *GelC03G01561*). *PSY* and *PDS* lead to the biosynthesis of (E/Z)-phytoene(15-*cis*-phytoene), 15,9′-tri-*cis*-phytofluene, and 9,15,9′-tri-*cis*-*ζ*-Carotene, *CCD7* catalyze 9-*cis*-*β*-Carotene to synthesize 9-*cis*-10′-Apo-*β*-carotenal. In our results, *PDS* (*GelC01G01668*) were significantly higher expressed in WM-F, HM-F when compared to LM-F, showing 75.66- and 32.75-fold for *PSY*, and 116.27-43.20-fold for *CCD7*, 16.65- and 14.33-fold for *PDS*, respectively; most of carotenoid biosynthesis genes had higher expression in flowers when compared to their flower stalks (Figure 4b, Supplementary Table S4).

### 2.5. Carotenoids That Contributed to the Pigmentation of G. elata Varieties

Carotenoids are a class of lipophilic pigments derived from the isoprenoid terpenoid pathway. According to spectrophotometric assay, quantitative analysis of the total carotenoid content at the blooming stage revealed relatively higher carotenoid accumulation in HM and WM flowers compared to LM (Figure S1). The comparative carotenoid profiling of *G. elata* samples were analyzed using a liquid chromatography–electrospray ionization–tandem mass spectrometry (LC-ESI-MS/MS, SCIEX) platform (MetWare Biotechnology Co., Ltd., Wuhan, China). The specific carotenoid metabolites from *G. elata* are listed in Table S5. Further targeted detection of carotenoids in the flowers and peduncles of different *G. elata* varieties revealed significant differences in the distribution and accumulation of these compounds across different tissue types and varieties. A total of 32 carotenoids were identified, comprising 5 carotenes and 27 xanthophylls. Among these, 10 carotenoids, including lycopene, α-carotene, and α-cryptoxanthin, exhibited significantly distinct distribution patterns in the flowers and flowers stalks of the three *G. elata* varieties (Figure 4c, Table S5). Analysis of differential metabolites showed that the *β*-branch pathway accumulates metabolites more predominantly than the α-branch pathway in the partitioning of metabolic flux within carotenoid biosynthesis along with distinct carotenoid accumulation profiles between WM, HM, and LM in both flowers and flower stalks. Fewer carotenoids were detected in flower stalks compared to flowers; at least 9 carotenoids, predominantly xanthophylls, were absent in flower stalks. Violaxanthin was the most abundant carotenoid in both WM and LM, reaching concentrations of 40.67 μg/g and 39.53 μg/g, respectively, but was lower in HM (29.22 μg/g). While zeaxanthin was only detected in LM flowers (0.43 μg/g). Three compounds, (*E*/*Z*)-phytoene (15-*cis*-phytoene), antheraxanthin, and capsanthin, were present at significantly higher levels in WM and HM, peaking in WM at 4.29 μg/g, 3.10 μg/g, and 0.70 μg/g, respectively. These concentrations were 2.41, 4.89, and 11.45 times higher than those in LM. (*E*/*Z*)-phytoene and capsanthin are red pigments, while antheraxanthin is yellow. These compounds likely contribute primarily to the red and orange-yellow coloration observed in WM and HM flowers and flowers stalks. In contrast, LM accumulated lower levels of these pigments. Xanthophylls, due to their hydroxyl groups, are often acylated with saturated fatty acids like lauric, myristic, and palmitic acid, forming esters in plants. This esterification preserves their antioxidant activity against superoxide anion, hydroxide radical, and singlet oxygen but significantly enhances their stability towards heat, UV light, and fruit storage/processing [11]. A total of 27 xanthophylls including 18 esters of *β*-cryptoxanthin, violaxanthin, and zeaxanthin were also detected in *G. elata*; 2 carotenes (lycopene and *α*-carotene) and 4 xanthophylls (8′-apo-beta-carotenal, *α*-cryptoxanthin, zeaxanthin palmitate, violaxanthin dioleate) were not detected in LM flowers.

### 2.6. Weighed Gene Co-Expression Network Analysis (WGCNA)

**Figure 5:**
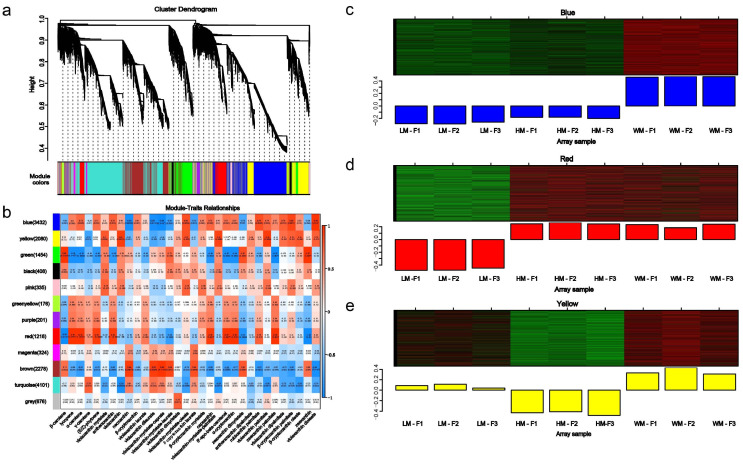
Weighted gene co-expression network analysis (WGCNA) of differentially expressed genes (DEGs) in *G. elata* flowers. (**a**) Cluster dendrogram of DEGs. (**b**) Correlation heat map between 12 gene modules and carotenoids content. (**c**–**e**) The relative expression of genes in the blue, red, and yellow modules related to carotenoids content. Green color indicates a lower relative expression level of genes, while red color indicates a higher relative expression level of genes.

In order to further explain the regulatory mechanisms of different carotenoids accumulation in *G. elata* flowers, WGCNA was conducted on differentially expressed genes to elucidate metabolite co-expression modules associated with different carotenoids’ accumulation and phenotypic traits in *G. elata*. DEGs are clustered into 12 modules, as shown in the hierarchical clustering tree (Figure 5a, Supplementary Table S6). These encompassed the following: blue (3432 genes), yellow (2080 genes), green (1454 genes), black (408 genes), pink (335 genes), green–yellow (176 genes), purple (201 genes), red (1218 genes), magenta (324 genes), brown (2278 genes), turquoise (4101 genes), and gray (676 genes). The correlations between the modules and the major carotenoids content were analyzed to identify the modules associated with flower colors and carotenoids’ accumulation (Figure 5b, Supplementary Table S7). Results indicated that the blue, red, and yellow modules had significant positive correlations with more carotenoids, while the green and brown modules had a significant negative correlation when compared to others. Especially, the red modules were highly significantly positively correlated with lycopene, α-carotene, (*E*/*Z*)-phytoene, capsanthin, 8′-apo-beta-carotenal, α-cryptoxanthin, zeaxanthin palmitate (correlation coefficient > 0.92, and *p* < 0.05). In addition, the yellow module was positively associated with violaxanthin myristate (r = 0.8), violaxanthin (r = 0.94), violaxanthin-myristate-oleate (r = 0.87), violaxanthin-myristate-palmitate (r = 0.91), and violaxanthin palmitate (r = 0.93), *β*-cryptoxanthin oleate (r = 0.81). The brown module was positively associated with neoxanthin (r = 0.92), violaxanthin-myristate-caprate (r = 0.96), violaxanthin-myristate-laurate (r = 0.89), *β*-cryptoxanthin laurate (r = 0.84), and zeaxanthin dimyristate (r = 0.81). The blue and yellow modules exhibited the higher expression level in WM-F, the blue, red, and yellow modules showed decreased or lower expression level in LM-F (Figure 5c–e).

### 2.7. MYB Transcription Factors Related to Carotenoids’ Synthesis in G. elata

**Figure 6:**
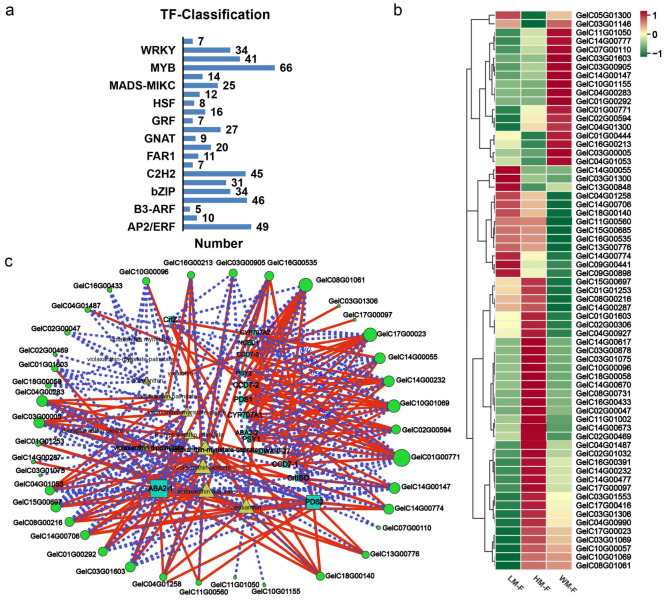
Differential transcription factor statistics in *G. elata* varieties. (**a**) The number of differential transcription factors in *G. elata* varieties. (**b**) The heat map of the differential expression profiles of MYB transcription factors in flowers. Red means up-regulated genes and green shows down-regulated genes; (**c**) The correlation analysis of MYB transcription factor, carotenoids biosynthesis genes, and carotenoids metabolites. The correlation network diagram is divided into three layers, the outermost genes are MYB transcription factors with green circle, the second layer is carotenoids with yellow color, and the last layer is carotenoids synthesis genes with blue color. The solid red lines and blue dashed lines connect the nodes representing MYB transcription factors to those of carotenoid biosynthesis genes and metabolites.

The differentially expressed transcription factors were also analyzed in three varieties of *G. elata*. A total of 524 differentially expressed transcription factors were identified, which mainly belonged to 23 TF families, of which MYB (66/524), AP2/ERF (49/524), bHLH (46/524), and C2H2 (45/524) were the top four highest families, followed by NAC (41/524), WRKY (34/524), and bZIP (34/524) family (Figure 6a). Numerous studies have indicated that transcription factors, especially MYB, play regulatory roles in the carotenoid biosynthetic pathway in plants. Especially, MYB TFs including *GelC01G00771*, *GelC03G00005*, and *GelC16G00535* were significantly up-regulated in flowers when compared to flower stalks of *G. elata*; *GelC03G01603* and *GelC14G00147* were higher expression in WM flower than LM and HM flower or flower stalks (Figure 6b, Supplementary Table S8). The content of carotenoids in the flowers was relatively higher, so our analysis focused on genes related to carotenoid biosynthesis and metabolism in flowers. Correlation analysis identified that many MYB transcription factors, such as *GelC01G00771*, *GelC08G01061*, *GelC14G00232*, *GelC17G00023, GelC04G00283*, and *GelC10G01069*, were strongly associated with *PDS*, *CrtISO*, *PSY*, *CCD7*, *CYP707A* genes, and main carotenoids (Figure 6c, Supplementary Table S9), combined with the results of the gene expression level and metabolic analyses, which suggested that MYB may play crucial regulatory roles in the carotenoid biosynthetic pathway of *G. elata*.

## 3. Discussion

### 3.1. Pigment Composition and Accumulation Govern Phenotypic Color Diversity

The coloration in plants is predominantly determined by the composition and abundance of pigments. In many species, the distinct hues of flowers, fruits, and other tissues arise from the tissue-specific and dynamic accumulation of carotenoids and/or anthocyanins. For instance, the yellow-, orange-, and red-peeled cashew apples (*Anacardium occidentale* L.) are primarily attributed to the dynamic and differential accumulation of carotenoid and anthocyanins [11]. Similar phenomenona have been documented in pineapple (*Ananas comosus* (L.) Merr.), Southwest iris (*Iris bulleyana* Dykes) [12], and *Lonicera japonica* [13]. Furthermore, comparative studies reveal that red-fleshed watermelon cultivars contain significantly higher levels of carotenoids such as lycopene and *β*-carotene than pink-fleshed varieties [14]. In roses, orange petals accumulate high levels of esterified xanthophylls derived from the *β*-branch pathway, whereas white petals are characterized by free carotenoids from the α-branch; notably, the content of violaxanthin myristate in orange petals was over 50-fold higher than in white petals [15]. A detailed study on cashew apples demonstrated that the carotenoids and their esters were the dominant pigments in yellow-, orange-, and red-peeled cashew apples, with orange varieties containing 3.01–3.19-fold higher concentrations of carotenoids than yellow and red ones, while the presence or absence of anthocyanins, especially 7-*O*-methylcyanidin 3-*O*-*β*-D-galactopyranoside, mainly contributed to red- or yellow-peeled cashew, yellow due to the absence of anthocyanins and orange resulting from elevated carotenoid levels [11]. A similar conclusion was also obtained according to our results, which demonstrate that the phenotypic variation among *G. elata* varieties is predominantly governed by pronounced differences in carotenoid composition and concentration, while the low abundance of anthocyanins is likely to exert a minor influence on the observed coloration.

The carotenoids and anthocyanins play a pivotal role in producing photosynthetic and photoprotective pigments, as well as visual and olfactory signals that attract animal pollinators and seed dispersers [1,2]. *G. elata* is a leafless orchid that obtains carbon through mycoheterotrophy. Unlike in photosynthetic plants, pigment synthesis occurs during bolting and flowering to provide floral coloration. Therefore, it is a reasonable inference that the vivid coloration of *G. elata* flowers, ranging from yellowish to reddish-brown, can be attributed to the targeted accumulation of pigments to attract pollinators. The higher accumulation of carotenoids in *G. elata* flowers results from this pigmentation presumably functioning as a visual signal to enhance pollinator visitation, thereby ensuring successful fertilization. Variation in pigment accumulation among plant ecotypes and geographical origins, largely driven by genetic diversity and environmental factors, is a well-documented phenomenon [1]. This pattern is consistently observed in *G. elata*, where different varieties exhibit distinct carotenoid profiles. Such divergence, which is evolutionarily correlated among germplasms, is similarly observed in other species, as seen in tomato genotypes with distinct colorations [16]. Comparative genomic analysis reveals that in the red-type tetraploid kiwifruit (*Actinidia arguta*), the bHLH transcription factor *AaBEE1* negatively regulates anthocyanin biosynthesis by binding to the G-box element within the *AaLDOX* promoter. Additionally, lineage-specific gene family contraction and expansion have been identified as major drivers of speciation in *A. arguta* [17]. Comparative genomics also indicate that gene family expansions are predominantly enriched in biosynthetic pathways of flavonoids, terpenoids, and phenylpropanoids in the pummelo (*Citrus maxima* or *Citrus grandis*), and contribute to the differential bioactive metabolites’ biosynthesis in *Citrus* [18].

Environmental factors such as light, plant hormones, and ethylene are involved in regulating carotenoid synthesis in plants. It has been reported that blue light promotes carotenoid accumulation in mango pulp by regulating the *MiCRY1-MiGAIP1-MiPIF5-MiAGL5* signaling pathway through crosstalk with plant hormones [19]. Previous research has shown that light signaling and plant hormones including ethylene (ET), abscisic acid (ABA), brassinosteroids (BR), and jasmonic acid (JA) positively regulate carotenoid metabolism during fruit ripening in tomatoes and others [20]. Gang et al. reported that gibberellin (GA) and prohydrojasmon (PDJ) applications effectively inhibit color development and carotenoid accumulation in mature citrus fruit; however, post-harvest treatment with indole-3-acetic acid (IAA) or 1-naphthaleneacetic acid (NAA) stimulated carotenoid accumulation in GA/PDJ-treated fruit through up-regulated carotenoid biosynthetic genes and suppressed catabolic gene expression to ensure the fruit quality; moreover, ethylene synthesis was induced by these treatments, which potentially facilitates carotenoid production through stimulating carotenogenesis in citrus fruit [21]. Carotenoid formation and accumulation are closely linked to plastid development, with light acting as a key regulator of both processes in plants [22]. During the bolting of *G. elata* tubers, light exposure presumably functions as a visual factor to promote carotenoid accumulation of inflorescence tissues through crosstalk with plant hormones.

### 3.2. The Regulation of Pigments’ Biosynthetic Pathway

The expression of structural genes directly drives the biosynthesis and accumulation of pigments, which ultimately shapes the diverse color phenotypes observed across plant species. Multi-omics analyses revealed that the significant correlations among the differential changes in color, the accumulation of carotenoids and the expression levels of structural genes in plants, which has been reported in tomato [16], pepper [23], Goji [24], citrus [25], *Gardenia jasminoides* [26], and so on. Research has shown that the differential expression of carotenoid biosynthetic pathway genes explains the distinct carotenoid accumulation patterns in the secondary phloem and xylem of carrot roots [27]. The diverse coloration phenotypes of Cassava (*Manihot esculenta* Crantz) storage roots correlate strongly with carotenoid content and gene expression level of carotenoid biosynthesis pathway [28]; this association enables rapid estimation of carotenoid levels based on phenotypic assessment [29]. In our results, *PDS*, *CCD7*, and *PSY* were significantly higher expressed in WM-F and HM-F compared to LM-F; a similar result was also found when compared to their flower stalks. These results indicated that these genes play an important role in carotenoids’ biosynthetic pathway in different *G. elata* varieties; meanwhile, it directly explained the differential accumulation of carotenoids in the flowers at the transcription level. Whether the correlation between this phenotype, metabolite levels, and gene expression can serve as a basis for distinguishing different *G. elata* varieties remains to be further investigated.

### 3.3. TFs Play a Critical Role in Orchestrating Color in Different Varieties of G. elata

Extensive research documents transcription factors (TFs) mediated regulation of biosynthetic pathways for plant metabolites including flavonoids, anthocyanins, and carotenoids [1]. Especially, R2R3-MYB transcription factors are increasingly recognized as key regulators in anthocyanins and carotenoids’ biosynthesis. The efficient engineering of the entire anthocyanin biosynthesis pathway was achieved by Sun et al. through tissue-specific expression of master transcriptional regulators *SlAN2-like* gene, which leads to high-level accumulation of anthocyanins in tomato’s peel and flesh [6]. It has been reported that the yellow spots on the lip of *Dendrobium devonianum* are primarily associated with carotenoid biosynthesis genes, while the purple coloration mainly links to anthocyanin biosynthesis genes, members of the MYB and bHLH transcription factor families (e.g., *DedeMYB305-1*, *DedebHLH104*), which exhibit strong positive correlations with these pigment synthesis genes, suggesting they likely serve as key regulatory TFs and controlling floral pigmentation patterning [30]. The evidence from kiwifruit (*Actinidia deliciosa*) showed that *AdMYB7* modulates carotenoid levels through its activation of *AdLCY-β* expression and suggests *MYB7* governs the accumulation of carotenoids and chlorophyll pigments by controlling the transcription of associated metabolic genes [31]. In the orange band of *Liriodendron tulipifera* petals, *LtMYB305* enhanced carotenoid synthesis in the petal’s central region by regulating *LtLCYB* expression, leading to specifically up-regulated carotenoid production along with flavonoid and chlorophyll biosynthesis being down-regulated [12]. In citrus fruit coloration, *CsMADS6* potentiates carotenoid biosynthesis by activating promoters of structural genes *LCYb1*, *PSY*, *PDS*, and *CCD1*, directly influencing carotenoids’ accumulation and fruit coloration [32]. Crocus transcription factors *CstMYB1* and *CstMYB1R2* coordinate carotenoid production through differential binding affinity to the *CCD2* or *PSY* promoters, ultimately governing chromophore formation and color presentation [33].

Transcription factors (TFs) orchestrate the biosynthesis of plant secondary metabolites by responding to environmental signals such as light, temperature, and hormones [34,35]. TFs orchestrate complex regulatory networks to exert their biological and metabolic functions [34]. A key mechanism involves the formation of MYB-bHLH-WD40 (MBW) complexes, which integrate these signals to regulate pigment accumulation and broader metabolic pathways at the transcriptional level, as systematically reviewed by Zheng et al. [35]. TF-mediated regulatory networks modulate the biosynthesis of diverse bioactive compounds. Xu et al. reported three transcription factors, *LhHB4*, *LhWRKY*, *LhMYBSPLATTER*, which belong to different TF families, forming an activation–inhibition module to regulate anthocyanin accumulation in Asiatic hybrid lilies [36]. According to our studies, several MYB TFs including *GelC01G00771*, *GelC03G00005* and *GelC16G00535* were selected and spatially expressed in different *G. elata* varieties. We hypothesize that these MYB TFs play crucial regulatory roles in the carotenoid biosynthetic pathway of *G. elata*. To validate this hypothesis, future studies should aim to identify the direct targets and elucidate the comprehensive regulatory network governed by these MYB TFs in *G. elata*.

## 4. Materials and Methods

### 4.1. Plant Materials and Sampling

The varieties of *G. elata* including *G. elata* Bl. f. elata (HM), *G. elata* Bl. f. g1auca, (WM) and *G. elata* Bl. f. viridis (LM) were purchased form Yunnan Hongyao Agricultural Development & Investment Co., Ltd. (Kunming, Yunnan, China). The selected tubers weighed between 150 g and 200 g, and were free from physical damage and visible pest or disease symptoms. The tubers were planted in the greenhouse at Hubei Agricultural University, Wuhan, Hubei Province, China (114.01° E, 30.00° N) starting on 23 February 2024. The tubers were grown in foam boxes (54 cm × 33 cm × 31 cm) containing clean river sand as the substrate. Throughout the cultivation period, environmental conditions were maintained at a constant temperature of 22 °C, with a 12 h light/12 h dark photoperiod and relative humidity of 60–70%. The flowers, flower stalks, and tubers from all three *G. elata* varieties were harvested during the full-bloom stage when approximately 70–80% of the flowers on the inflorescence had opened. The sampling was performed between 26 March and 10 April 2024. All collected samples were immediately frozen in liquid nitrogen and stored at −80 °C until further use.

### 4.2. Metabolites Analysis

#### 4.2.1. Targeted Metabolomics Analysis for Anthocyanins

Metabolite extraction was performed as described: 50 mg freeze-dried powder underwent mixing with 0.5 mL methanol/water/hydrochloric acid (799:200:1, *v*/*v*/*v*), then ultrasound for 30 min with vortexing for 30 s at 10 min intervals, and the extract was centrifuged at 4 °C (12,000× *g*, 3 min). The resulting supernatant was filtered through a 0.22 μm microporous membrane prior to UPLC-MS/MS analysis. Anthocyanin contents were quantified via the AB Sciex QTRAP 6500 LC-MS/MS (SCIEX, Framingham, MA, USA) platform provided by MetWare (http://www.metware.cn/). UPLC-ESI-Q TRAP-MS/MS conditions and parameters were referenced from established methods. Analysis employed the scheduled multiple reaction monitoring (MRM) approach established by Fraga et al. [37]. Compound annotation integrated MetWare’s MWDB repository with public databases including MassBank, KNAPSAcK, HMDB, MoTo DB, and METLIN. All analyses incorporated triplicate technical replicates. Data processing was performed using Analyst 1.6.3 and MultiQuant 3.0.3 software. Standard curves were constructed based on the concentration and peak area of each anthocyanin.

#### 4.2.2. The Metabolic Analysis of Carotenoids

Total carotenoid content was detected according to the spectrophotometric method. The dry powder of *G. elata* (0.1 g) was extracted with 1.0 mL of methanol using ultrasonic extraction at 400 W and 25 °C for 30 min, followed by centrifugation at 25 °C and 12,000× *g* for 5 min. Then, the supernatant was collected for total carotenoid content analysis. The absorbance of the above supernatant was measured at 662, 645, and 470 nm via spectrophotometry, and triplicate biological replicates was performed to ensure the reproducibility.

For the targeted metabolomics analysis, freeze-dried samples were finely ground into powder following extraction by 0.5 mL of a mixture composed of hexane, acetone, and ethanol, (1:1:1, *v*/*v*/*v*). The samples were vortexed for 20 min at room temperature, followed by centrifugation at 12,000 rpm for 5 min at 4 °C, and the supernatant was collected. This process was repeated, and the combined supernatants were evaporated to dryness and reconstituted with 100 μL of dichloromethane. Finally, the samples were passed through a 0.22 μm filter membrane to prepare them for subsequent analysis. The mixed solutions were utilized as QC samples. Carotenoid-targeted metabolomics was analyzed by MetWare (Wuhan, China). The LC-MS analysis was carried out using an advanced UPLC-APCI-MS/MS system, which included an ExionLC™AD for UPLC (SCIEX, USA) and an Applied Biosystems 6500 Triple Quadrupole for mass spectrometry (SCIEX, USA). A YMC C30 column (3 μm, 100 mm × 2.0 mm) was utilized for chromatographic analysis. The samples were gradient eluted using mobile phase A (a mixture of methanol and acetonitrile in a 1:3 *v*/*v* ratio, containing 0.01% BHT and 0.1% formic acid) and mobile phase B (methyl tert-butyl ether with 0.01% BHT). The flow rate was set at 0.8 mL/min, the column temperature was maintained at 28 °C, and an injection volume of 2 μL was used. Mass spectrometry was conducted at an Atmospheric Pressure Chemical Ionization Source (APCI) at 350 °C, with a curtain gas pressure of 25 psi and an RF of less than 0.7 Da. The mass spectrometry data were qualitatively analyzed using the Metware Database (MWDB) created from the standards. For quantitative analysis, Multiple Reaction Monitoring mode on a triple quadrupole mass spectrometer was employed. Data processing was performed using Analyst 1.6.3 and MultiQuant 3.0.3 software. Standard curves were constructed based on the concentration and peak area of each carotenoid.

### 4.3. RNA Extraction and Library Sequencing

RNA was extracted from *G. elata* samples for library sequencing. Total RNA was isolated from distinct floral samples using TRIzol reagent (Invitrogen, Carlsbad, CA, USA) according to manufacturer specifications. RNA concentration/purity measurements employed a NanoDrop ND-1000 spectrophotometer (NanoDrop Technologies, Wilmington, DE, USA), while the RNA integrity verification utilized the Agilent Bioanalyzer 2100 system (Agilent Technologies, Santa Clara, CA, USA). For transcriptome analysis, mRNA enrichment via oligo (dT)-coupled magnetic beads preceded cDNA synthesis with SuperScript™ II Reverse Transcriptase (Invitrogen, Carlsbad, CA, USA). Sequencing adapters were ligated prior to library preparation and high-throughput sequencing on an Illumina Novaseq™ 6000 system (LC-Bio, Hangzhou, China).

### 4.4. Transcriptome Data Analysis

To investigate the different biosynthetic regulation of metabolites in three varieties of *G. elata*, RNA-seq analysis was conducted using the Illumina HiSeq 2000 platform (San Diego, CA, USA). Initial processing of raw sequencing data utilized fastp v0.19.3 for quality control, eliminating low-quality reads and sequences with excessive N-content. Clean RNA-seq reads were then aligned to the *G. elata* reference genome using HISAT2 v2.1.0. Transcript assembly and novel gene prediction were conducted with StringTie v1.3.4d, while transcript abundance quantification employed the FPKM (fragments per kilobase of transcript per million fragments mapped) metric through featureCounts v1.6.2. Differential expression screening between different groups applied the DEGseq2 R package (v1.24.0), with significance thresholds set at |log_2_FC| ≥ 1 and false discovery rate (FDR) ≤ 0.05. Annotated DEGs underwent functional characterization via GO and KEGG enrichment analyses (corrected *p* ≤ 0.05).

### 4.5. Integrative Analysis of Transcriptomic and Metabolomic Profiles

Genes differentially expressed (DEGs) related to anthocyanin biosynthesis, along with the corresponding transcription factors (TFs), exhibited correlation with 15 differentially accumulated anthocyanin metabolites (DAMs). Integrative analysis involved calculating FPKM values for genes and metabolite abundance levels, with DEG screening criteria set at |r| > 0.8 and metabolite significance defined by |r| > 0.9 and a *p*-value < 0.05. Interactive networks linking anthocyanin-related DEGs and DAMs were visualized using an online platform (https://cloud.metware.cn).

### 4.6. Data Processing and Statistical Analysis

Statistical analyses including Pearson correlation, principal component analysis (PCA), hierarchical clustering (HCA), and a comprehensive quality evaluation model were performed using SPSS software (Version: 22.0). Orthogonal partial least-squares discriminant analysis (OPLS-DA) was conducted utilizing the online platform (https://cloud.metware.cn). Statistical analyses were executed in SPSS software (SPSS, Chicago, IL, USA), employing Student’s *t*-test and one-way ANOVA with Tukey’s post hoc comparison, and a *p*-value of less than 0.05 was deemed statistically significant. Distinct lowercase letters above error bars signify statistical significance (*p* < 0.05) according to one-way ANOVA (Tukey) analysis. To address multiple comparison artifacts, *p*-values underwent Benjamini–Hochberg false discovery rate (FDR) correction, with significance defined at q < 0.05.

## 5. Conclusions

This study investigated pigments’ metabolite profiles, especially carotenoid biosynthesis in three varieties of *G. elata*. The differential metabolomic and transcriptomic profiling of these three *G. elata* varieties were exhibited. The metabolomic analysis showed that the differential accumulation of carotenoids may play a more prominent role in various colorations in *G. elata* varieties. The results revealed that violaxanthin, (*E*/*Z*)-phytoene, antheraxanthin, and capsanthin were the major carotenoids, which differentially contributes to the pigment accumulation in *G. elata*. The candidate genes including *PSY*, *CCD*, *UGT*, and *PDS* genes were selected and considered as critical genes for the differential accumulation of carotenoids’ synthesis in *G. elata* varieties. Combined with multi-omics analysis, our results indicated transcription factors such as MYBs likely relate to the carotenoids biosynthesis of *G. elata*. The gene functions of transcription factors and their regulatory mechanisms governing metabolic biosynthesis in *G. elata* remain to be elucidated. Totally, this study enhances the current understanding of pigments’ metabolic profiles of *G. elata*. Our study contributes valuable insights into the molecular mechanisms of pigment biosynthesis underlying *G. elata*; these findings also provide valuable guidance and genetic resource for future nutritional biofortification and molecular breeding in *G. elata*.

## Data Availability

The original contributions presented in this study are included in the article/Supplementary Materials. Further inquiries can be directed to the corresponding author.

## Supplementary Materials

The supporting information can be downloaded at: https://www.mdpi.com/article/10.3390/ijms262411839/s1.
