# Supplementary material for: Integrated Omics Analysis Revealed the Differential Metabolism of Pigments in Three Varieties of Gastrodia elata Bl
Source: Int J Mol Sci. 2025 Dec 9;26(24):11839. doi: 10.3390/ijms262411839 (PMC12733335; doi:10.3390/ijms262411839)
Supplement: Supplementary file 1 [file ijms-26-11839-s001.zip › Figure. S1.pdf]

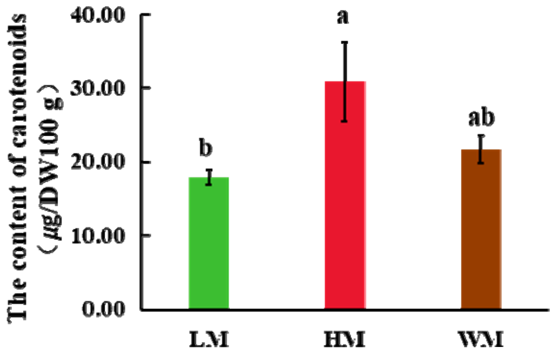

Figure S1. The total carotenoid contents of three *G. elata* varieties. The data represent the means in triplicate (n = 3); Different superscript letters indicate significant differences ($p < 0.05$).
