# Supplementary material for: Integrated Omics Analysis Revealed the Differential Metabolism of Pigments in Three Varieties of Gastrodia elata Bl
Source: Int J Mol Sci. 2025 Dec 9;26(24):11839. doi: 10.3390/ijms262411839 (PMC12733335; doi:10.3390/ijms262411839)
Supplement: Supplementary file 1 [file ijms-26-11839-s001.zip › Supplementary Materials list.pdf]

Table S1. DEGs of transcriptome sequencing in *G. elata*.

Table S2. The different expression of genes in phenylpropanoid-anthocyanin biosynthesis pathways.

Table S3. Metabolome Profiling of anthocyanins in *G. elata* varieties.

Table S4. The different expression of genes in carotenoids biosynthesis pathways.

Table S5. Metabolome Profiling of carotenoids in *G. elata* varieties.

Table S6. The co-expressed network of mian modules.

Table S7. The correlation between carotenoids and DEGs.

Table S8. The differential MYB transcription factors in *G. elata* varieties.

Table S9. The correlation of genes and carotenoids metabolites.

Figure S1. The total carotenoid contents of three *G. elata* varieties.
